# Catheter-Associated Urinary Tract Infection in Intensive Care Unit Patients at a Tertiary Care Hospital, Hail, Kingdom of Saudi Arabia

**DOI:** 10.3390/diagnostics12071695

**Published:** 2022-07-12

**Authors:** Mohd Saleem, Azharuddin Sajid Syed Khaja, Ashfaque Hossain, Fahaad Alenazi, Kamaleldin B. Said, Soha Abdallah Moursi, Homoud Abdulmohsin Almalaq, Hamza Mohamed, Ehab Rakha, Sunit Kumar Mishra

**Affiliations:** 1 Department of Pathology, College of Medicine, University of Hail, Hail 55211, Saudi Arabia; m.saleem@uoh.edu.sa (M.S.); kbs.mohamed@uoh.edu.sa (K.B.S.); s.moursi@uoh.edu.sa (S.A.M.); 2 Department of Medical Microbiology and Immunology, RAK Medical and Health Sciences University, Ras Al Khaimah 11172, United Arab Emirates; ashfaque@rakmhsu.ac.ae; 3 Department of Pharmacology, College of Medicine, University of Hail, Hail 55211, Saudi Arabia; fs.alenazi@uoh.edu.sa; 4 Hail Health Cluster, King Khalid Hospital, College of Pharmacy, King Saud University, Riyadh 11451, Saudi Arabia; halmalaq@moh.gov.sa; 5 Department of Anatomy, College of Medicine, University of Hail, Hail 55211, Saudi Arabia; h.alamin@uoh.edu.sa; 6 Microbiology Department, King Khalid Hospital, Hail 55421, Saudi Arabia; ehabrakha@yahoo.com; 7 Clinical Pathology Department, Faculty of Medicine, Mansoura University, Mansoura 35516, Egypt; 8 Ayurveda Consultant, King George’s Medical University, Lucknow 226003, India; drsunitmishra@gmail.com

**Keywords:** catheter-associated urinary tract infection (CAUTI), *Klebsiella pneumoniae*, hospital-acquired infections, *Proteus mirabilis*, *Pseudomonas aeruginosa*, antibiogram

## Abstract

Catheter-associated urinary tract infections (CAUTIs) are some of the most common hospital-acquired infections (HAIs). Prolonged hospitalization, invasive devices such as catheters, and irrational use of antimicrobial agents are believed to be the major causes of high rates of HAIs. Infections such as pyelonephritis, urethritis, cystitis, and prostatitis are the main concerns in catheterized ICU patients. In these cases, Gram-negative bacteria are the most common bacteria. The present study was undertaken to determine the frequency, antibiograms, disease pattern, and risk factors involved in providing an advocacy recommendation to prevent CAUTI. A total of 1078 patients were admitted to the hospital ICU, out of which healthcare-associated infection was reported in 316 patients. CAUTI was reported only in 70 patients. *Klebsiella pneumoniae* (20%) was the predominant isolate, with *Serratia* (3%) and *Providencia* (3%) species being the least common isolates in this study. The present study provides CAUTI incidence rates in a tertiary care hospital in Hail, Saudi Arabia. Furthermore, information on the risk factors of common associated CAUTI causative organisms and their antibiogram patterns are also presented. This study provides vital information that can be used to formulate an effective antibiotic stewardship program that can be implemented throughout the kingdom.

## 1. Introduction

Catheter-associated urinary tract infections (CAUTIs) are the most common hospital-acquired infections (HAIs). Prolonged hospitalization, use of invasive devices such as catheters, and irrational use of antimicrobial agents are believed to be the major causes of higher rates of HAIs [1]. It is estimated that infections in acute care units in hospitals represent more than 30% of annual infections [2]. Central line-associated bloodstream infections (CLABSIs) are the most common hospital-acquired infections related to invasive devices [3], followed by catheter-associated urinary tract infections (CAUTIs) and ventilator-associated pneumonia (VAP) [4].

During hospitalization, indwelling urethral catheters account for about 80% of urinary tract infections [3]. However, catheter placement is not the main reason for the development of UTIs. Catheters may facilitate colonization of the urinary bladder due to poor catheter placement, prolonged catheterization, poor aseptic technique, poor hand hygiene, and poor asepsis of the urethral orifice opening. Hence, catheters are the most common source of infection [5].

For each patient, the test result and frequency of a urinary tract infection can differ significantly, depending on age, comorbidities, and socioeconomic status. Gram-negative bacteria, such as *Escherichia coli*, *Klebsiella* spp., *Proteus mirabilis*, *Pseudomonas aeruginosa*, and *Citrobacter* spp., are the predominant isolates in urinary tract infections. Gram-positive bacteria such as *Staphylococcus aureus* and *Enterococcus species* are the most common [6,7]. On the other hand, secondary hospital-acquired bloodstream infections may occur post-catheter-associated urinary tract infections, as 17% of nosocomial bacteremia emerges from urinary tract infections, with an associated mortality of 10% [8]. Moreover, asymptomatic bacteriuria (presence of a significant bacterial count, i.e., >10^5^ CFU/mL in a well-collected urine sample with aseptic precautions from a patient with no signs or symptoms of urinary tract infection) is very common in clinical practice [9]. However, it is associated with a low number of sequelae and low morbidity, and in the majority, it is self-limiting except in pregnant women where asymptomatic bacteriuria should be treated. The most common signs and symptoms of urinary tract infections are fever, flank pain, suprapubic pain, dysuria, urinary urgency, and hematuria. Long-term hospitalization inferable to device-related infections should be an avoidable situation; moreover, there is an increase in treatment costs and risk of lethality for patients whenever it occurs. The present study was undertaken to determine the frequency, antibiograms, disease pattern, and risk factors involved and to offer an advocacy recommendation for preventing CAUTIs.

## 2. Materials and Methods

### 2.1. Study Design, Duration, and Settings

The present study was a cross-sectional hospital-based study conducted in the division of Microbiology, Department of Pathology, College of Medicine, University of Hail, Hail, Kingdom of Saudi Arabia (KSA), from January 2020 to December 2021. The patient information was obtained from the ICU of King Khalid Hospital, Hail, Saudi Arabia.

### 2.2. Study Population

This study included adult patients ≥ 18 years who were admitted to the ICU during the study period with medical conditions including urinary tract infections (UTIs) and were catheterized using a Foley catheter. Patients who were admitted with positive urine cultures before catheterization were excluded from the study.

### 2.3. Ethical Considerations

This research was performed after obtaining approval from the Ethics Committee, Research Deanship, University of Hail (H-2020-236, letter number 23561/5/42; IRB Registration Number with KACS: H-08-L-074). Before enrolling in this study, all the participants were requested to sign informed consent forms. All the study-related information and data were secured by using unique identifying numbers to ensure confidentiality throughout the study.

### 2.4. Study Tools and Data and Sample Collection

Patient information, including demographics (age, sex), clinical data, type and cause of admission, risk factors, comorbidities, causes of urinary catheterization, antibiogram, and outcome of CAUTI management, was collected from the medical record files. No personally identifiable information was retrieved. 

For quantitative microbiological culture, 10 mL of midstream urine sample was collected from the catheter tube using a sterile disposable syringe in a sterile universal container from each patient. While collecting the urine sample, all mandatory aseptic precautions were taken as the site of aspiration in the catheter tube was primarily cleaned using 70% ethanol. The catheter tube was clamped proximally to the urethral or suprapubic opening to collect freshly voided urine. Freshly collected samples were then sent to the microbiology department in a cold storage transfer container without delay.

### 2.5. Urine Culture and Microscopic Examination

Direct wet mount of uncentrifuged urine samples was conducted to determine pyuria and bacteriuria under a high-power field. Urine samples were then directly inoculated on a BD Cysteine-lactose-electrolyte-deficient (CLED) agar plate using a standard-dimension disposable plastic inoculating loop with an internal diameter of 3.26 mm. The dish was then incubated at 37 °C for 16–18 h in an incubator. The number of colonies was counted using a microprocessor colony counter, and this number was used to calculate the number of viable bacteria per mL of urine. Thus, if 0.001 mL of urine yields 100 colonies, the count per mL will be 10^5^, or just indicative of significant bacteriuria. Hence, with significant growth, ≥10^5^ CFU/mL, isolates were identified to possible species level by the conventional method using standard biochemical media (catalase, oxidase Invitrogen Amplex Red, slide coagulase Thermo Scientific Oxoid, Indole Kovac’s reagent, Methyl red CH-METHRED, Citrate utilization test, Christensen’s urease agar HIMEDIA M1125-500G, TSI agar CHEMsolute, Bile esculin HIMEDIA M972A-500G) as per the requirements for both Gram-negative and Gram-positive bacteria as reported in earlier studies [10,11]. Further, this was confirmed by the BD Phoenix M50 system (BD Diagnostic Systems, Oxford, UK) using antibiotic susceptibility patterns according to the manufacturer’s recommendations.

Preliminary identification was conducted using the conventional method. In contrast, the automated method was based on chromogenic and fluorogenic reactions, and the AST was based on turbidimetry and redox reactions to determine each antibiotic’s minimal inhibitory concentration (MIC) as per the CLSI guidelines 2020 [12].

## 3. Results

**Figure 1:**
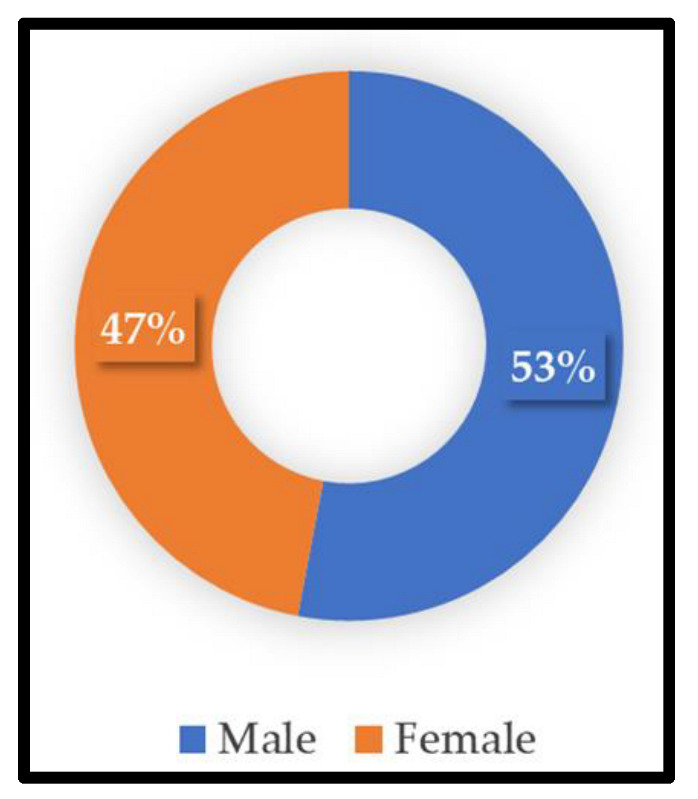
Gender-specific distribution of patients with CAUTI in the study participants.

A total of 1078 patients were admitted to the hospital’s intensive care unit, of which HAI was reported in 316 patients. Among these, CAUTI was reported only in 70 patients. Table 1 shows patients’ demographics reported with CAUTI. The most common age group seen with CAUTI infection was 70–80 years (*n* = 21, 30%), followed by 60–70 years (*n* = 16, 23%), and 50–60 years (*n* = 12, 17%), with the least common age group being 30–40 years (*n* = 2, 3%). Significantly more male patients had CAUTI compared to female patients, with a ratio of 1.12 (37/33; Figure 1). Under the BMI distribution, 28 patients were overweight (40%), while 12 were obese (17%). 

Table 2 shows that most patients had urethral catheterization (51; 73%) with a few suprapubic catheterizations (19; 27%). The 16 Fr catheter size was the most common (*n* = 42, 60%), with 18 Fr being the least common size (17%). The majority of the patients with long-term indwelling urethral catheterization (IUC) had a significantly higher prevalence of CAUTI (37; 53%) than those with short-term IUC (13; 13%). Significantly, the patients with long-term IUC had a significantly higher prevalence of benign prostatic hyperplasia (BPH) (37; 53%) than the patients with short-term IUC. The majority of patients with IUC had no other comorbidities (*n* = 42, 60%).

Table 3 presents the symptoms that persisted in the study group, where fever was the most common symptom (*n* = 23, 33%), followed by flank pain (*n* = 14, 20%), urinary urgency (*n* = 9, 13%), and suprapubic pain (*n* = 8, 11%), with hematuria (*n* = 3, 4%) as the least recorded symptom.

**Figure 2:**
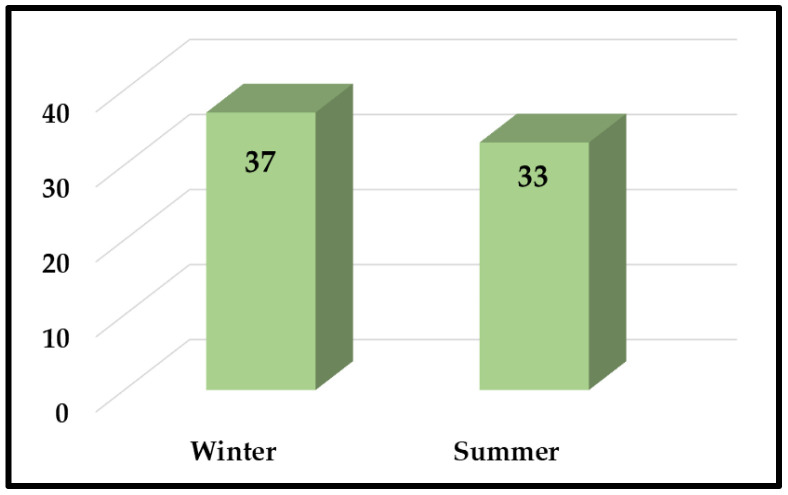
Number of uropathogens from CAUTI patients during summer and winter.

Table 4 shows the month-wise distribution of bacterial isolates from CAUTIs in the ICU patients. The months were categorized into two seasons: summer (March to August) and winter (September to February). Figure 2 shows that more cases of CAUTI were seen during winter (*n* = 37, 53%) than in summer (*n* = 33, 47%).

In this study, the most common bacterial species found associated with CAUTI was *Klebsiella pneumoniae* (*n* = 15, 21%), followed by *Proteus mirabilis* (*n* = 12, 17%), *Pseudomonas aeruginosa*, and *Enterococcus faecalis* (*n* = 9, 13%) (Table 5). We found two uncommon bacterial isolates in our setting: *Serratia* (*S. marcescens* (*n* = 5, 7%), *S. macalane* (*n* = 2, 3%), and *S. plymuthica* (*n* = 2, 3%)), and *Providencia stuartii* (*n* = 2, 3%).

The antimicrobial susceptibility pattern of *Klebsiella pneumoniae* (Table 6) showed high sensitivity towards amikacin (100%), followed by colistin, gentamicin, and imipenem (86.7%). In contrast, with cefepime, levofloxacin, meropenem, piperacillin/tazobactam, and sulfamethoxazole/trimethoprim, only 66.7% sensitivity was found. With ciprofloxacin, ceftazidime, teicoplanin, and amoxiclav, 53.3% sensitivity was observed. High resistance of *Klebsiella pneumoniae* was observed for ceftriaxone, mupirocin, and tigecycline (80%), followed by nitrofurantoin, aztreonam, cefuroxime, and cephalothin (66.7%). In our setting, empirical therapy was based on epidemiological data, antibiograms, and hospital guidelines where broad-spectrum antibiotics were avoided in the first line of antimicrobial treatment. For *Klebsiella pneumoniae*-like bacteria, amoxicillin/clavulanic acid and nitrofurantoin were prescribed as the first line of therapy. In our study, nitrofurantoin was prescribed as the first choice of drug for uropathogens, but we reported high resistance to it (66.7%). Furthermore, we found less susceptibility against ciprofloxacin for *K. pneumoniae* (46.7%), which might be because ciprofloxacin is one of the most widely administered antibiotics in our setting.

The susceptibility pattern against *Proteus mirabilis* was also investigated (Table 7). It was highly sensitive to nitrofurantoin and cefoxitin (100%), followed by amikacin, piperacillin/tazobactam, ertapenem (83.3%), amoxicillin/clavulanic acid (58.3%), and meropenem (50%). High resistance of *Proteus mirabilis* was also observed for cefepime, colistin, levofloxacin, sulfamethoxazole/trimethoprim, daptomycin, linezolid, tetracycline, vancomycin, erythromycin, and TZP (83.3%), followed by mupirocin (58.3%). *Pseudomonas aeruginosa* was highly sensitive to amikacin, cefepime, colistin, ciprofloxacin, and ceftazidime (100%), followed by gentamicin, imipenem (77.8%), levofloxacin, and meropenem (55.6%; Table 7). It was highly resistant against piperacillin/tazobactam, teicoplanin, cefoxitin, aztreonam, sulfamethoxazole/trimethoprim, TZP (77.8%), and imipenem (66.7%). *Enterococcus faecalis* was highly sensitive to daptomycin (100%), followed by vancomycin, ampicillin, moxifloxacin (77.8%), nitrofurantoin, amoxicillin/clavulanic acid, sulfamethoxazole/trimethoprim, and linezolid (66.7%), whereas it showed only 55.6% sensitivity to ciprofloxacin. This bacterium is highly resistant to ceftazidime, teicoplanin, and fusidic acid (77.8%; Table 7).

## 4. Discussion

Urinary tract infections are some of the most common clinical conditions in human medicine, which affect a wide range of discrete population groups irrespective of age and gender. Long-term UTIs may result in developing chronic diseases. They also dramatically affect the socioeconomic lives of infected individuals, contributing largely to the increased intake of antibiotics. Community-acquired and healthcare-associated urinary tract infections should be considered a serious public health issue and an economic burden. The etiological agents of UTIs show diversity, especially in hospital settings, where prolonged catheterization and immunosuppressive drugs are used.

In this study, 1078 patients admitted to the ICU were examined, out of which HAI was reported in 316 (29.3%) patients. CAUTI was reported only in 70 (22%) patients. Our result is comparable to the prevalence rate of other studies such as H Bizuayehu et al. (21%) and Vinoth et al. (20%) [13,14]. Irrational use of antibiotics, gender, extremes of age, length of ICU stay, use of immunosuppressive drugs, and indwelling urethral catheterization have been considered risk factors for the increased incidence of CAUTI in ICU patients. In our study, a higher number of CAUTI cases were found to be associated with extreme age group patients (52.8%, 37/70), who were between 60 and 80 years of age. Several studies suggest CAUTI cases increase with advancing age [15,16,17]. In our research, more male patients were recorded with CAUTI than females, with a ratio of 1.12 (37/33), which is similar to the findings of other authors (1.89, 144/76; 1.67, 102/61) [14,18]. In our study, female gender was not a risk factor for CAUTI. In contrast, other studies concluded that females had a stronger predisposition to CAUTI [19,20,21]. Possibly, this result can be explained by the lower number of female patients in our study. In the present study, most patients presented only with a fever (*n* = 23, 33%), and there was a positive association between the fever and CAUTI. Fever is a particularly common symptom among critically ill patients, and evaluation for CAUTI should be conducted if fever is present in catheterized patients [8].

Significantly, the patients who had long-term IUC were older than those who had short-term IUC; in older men, there is an underlying physiological change in the prostate gland (due to benign prostatic hyperplasia), as seen in this study (*n* = 37, 53%), which subjects them to the development of UTIs. Numerous studies reported that the longer the catheter remains indwelling inside the urethra, the higher the rate of bacteria colonizing the urinary bag and ascending in the drainage tubing towards the bladder, resulting in CAUTI [22,23]. Using the Society for Healthcare Epidemiology of America/Infectious Diseases recommendation, Abdul Muttalib DA et al., 2013, in a Saudi Arabian study, concluded that removing the catheter as soon as possible reduced CAUTI [24]. According to a study by Tasseau et al., the risk of CAUTI rose from 19% to 50% when the catheterization duration increased from 5 to 14 days. They also reported that each day of catheterization increased the risk of CAUTI development by 5%, depending on the most frequent species and its antibiogram. Virtually all patients were found colonized by day 30 in a separate report [25], which was found to be a result of the urinary catheter and disruption of host defense mechanisms, as microbes were able to attach and eventually form biofilms [26,27,28].

Regarding the catheter size, a significant number of patients used the 16 Fr size in our study. It is recommended that a smaller-size catheter be used to provide better drainage with a 5 mL balloon inflated with 10 cc sterile water to ensure balloon symmetry for better clearance of the urinary bladder. Irreparable destruction caused to the urethra and bladder neck by using large-size catheters sometimes causes bladder spasms. It also causes hindrance in drainage of the peri-urethral gland, which can enhance the risk of infection [29]. The prevalence of CAUTI in this study was higher in comparison to other studies by Podkovik S et al. (8.5%) and Getliffe K et al. (4.76%) [30,31].

In this study, 100% (70/70) of CAUTIs were due to bacterial etiologies. Of 70 bacterial isolates, 61 (87%) were Gram-negative bacteria and 9 (13%) were Gram-positive bacteria. Among the Gram-negative bacterial isolates, *Klebsiella pneumoniae* was the predominant isolate (*n* = 15, 21%), followed by *Proteus mirabilis* (*n* = 12, 17%), *Pseudomonas aeruginosa* (*n* = 9, 13%), and *E. coli* (*n* = 7, 10%), with *Serratia* and *Providencia* species being the least common (3%). Of the Gram-positive bacteria, *Enterococcus feacalis* (*n* = 9, 13%) was the only isolate reported in this study. Other authors also reported Gram-negative bacteria as the most common etiological agents of CAUTI. However, the predominant bacterial isolate reported in our study was contradictory to many other studies that reported *E. coli* as the most common isolate, followed by *Klebsiella pneumoniae* [32,33,34]. Moreover, another study conducted by Shiva et al. reported that 69.2% of CAUTIs had a bacterial origin, and 30% were due to pathogenic yeast [18]. Although *Candida* is not on the CDC’s list of pathogens that cause CAUTI, a high proportion of *Candidia* cannot be ignored. 

Resistance to antimicrobial agents has been reported since the beginning of their use and is an emerging global concern. In our study, all Gram-negative isolates showed great variation in sensitivity patterns. *Klebsiella pneumoniae* showed extreme resistance toward ceftriaxone, tigecycline, and TEG (80%), whereas 66.7% of the isolates were resistant to nitrofurantoin, aztreonam, cefuroxime, and cephalothin. Moreover, it showed high sensitivity against amikacin (100%), gentamicin (86.7%), and imipenem (86.7%). These patterns are in agreement with the reports of other authors [29,35]. Against aminoglycosides, we noted that most of the *K. pneumoniae* bacteria were far more susceptible to amikacin (100%) than to gentamicin (86.7%).

For *Proteus mirabilis*, absolute susceptibility against nitrofurantoin cefoxitin was reported with high resistance against sulfamethoxazole/trimethoprim, levofloxacin, linezolid, tetracycline, vancomycin, and TZP (83.3%). The third most common isolate of this study, *Pseudomonas aeruginosa*, was highly sensitive toward colistin, amikacin, and ciprofloxacin (100%). This result is in line with another similar study by Alzahrani et al., who reported 100% sensitivity for colistin but with a descending sensitivity for amikacin (70%) and ciprofloxacin (60%) [32]. Ciprofloxacin is a medication that is widely used to treat UTIs. In our setting, we tested this antibiotic against 52 isolates and found that 35 (67%) were sensitive, while only 17 (32.6%) were resistant. This finding suggests that we can consider ciprofloxacin in the first-line treatment of UTI patients.

Multidrug resistance, as one of the current life-threatening issues in medicine, has been increasing significantly globally [36] and is more frequent in healthcare-associated infections than community-acquired infections. In Gram-negative bacteria, extremely high MDR is being reported. The most concerning part of this study was that high counts of Gram-negative bacteria developed MDR strains at varying frequencies. The wide distribution of bacteria from the Enterobacteriaceae family in our study area with easy exchange of plasmids encoding for ESBL and other resistance codons that code for resistance to other classes of antibiotics contributed to the emergence of MDR prevalence in the present study.

## 5. Conclusions

The present study provided CAUTI incidence rates in King Khaled Hospital in Hail, Saudi Arabia. A similar situation may exist in other government hospitals in Saudi Arabia. Furthermore, information on the risk factors of common associated CAUTI causative organisms and their antibiogram patterns were also presented. This study has significant clinical implications for patient treatment. It provides vital information that can be used to formulate an effective antibiotic stewardship program that can be implemented throughout the kingdom.

## Figures and Tables

**Table 1:** Age- and gender-specific frequency of CAUTI in the study participants.

Age Group (Years)	Number of Patients	Percentage (%)
20–30	5	7
30–40	2	3
40–50	5	7
50–60	12	17
60–70	16	23
70–80	21	30
80–90	9	13
**Sex**		
Male	37	53
Female	33	47
**BMI**		
Underweight	11	16
Normal weight	19	27
Overweight	28	40
Obese	12	17

**Table 2:** Clinical characteristics of patients with CAUTI in the study participants.

Patient Characteristics	Type of Catheter (*n* = 70)	Percentage (%)
Urethral type	51	73
Suprapubic type	19	27
**Cather size (French size) Fr**		
14 Fr	16	23
16 Fr	42	60
18 Fr	12	17
**Duration of indwelling urethral catheterization (days)**		
0–14	13	18.5
15–30	20	28.5
>31	37	53
**Implication for catheterization**		
Urine retention	2	3
Urethral stricture	9	13
Benign prostatic hyperplasia	37	53
Urinary incontinence	20	27
Others	2	3
**Comorbidity factors**		
With comorbidity	28	40
Without comorbidity	42	60

**Table 3:** Rate and frequency of clinical symptoms in CAUTI patients.

Symptoms	Number of Patients (*n* = 70)	Percentage (%)
Only fever	23	33
Flank pain	14	20
Suprapubic pain	8	11
Fever; suprapubic pain	6	9
Dysuria	7	10
Urinary urgency	9	13
Hematuria	3	4
Confusion	0	0

**Table 4:** Month-wise distribution of bacterial isolates from CAUTI patients.

Month	Isolate Number (*n*)	Percentage (%)
January	2	3
February	5	7
March	5	7
April	7	10
May	2	3
June	7	10
July	7	10
August	5	7
September	14	20
October	9	13
November	2	3
December	5	7
**Total**	**70**	**100**

**Table 5:** Rate of bacterial species identified from CAUTI patients.

Bacteria	CAUTI	Percentage (%)
*Acinetobacter baumannii*	5	7
*Escherichia coli*	7	10
*Enterococcus faecalis*	9	13
*Klebsiella oxytoca*	2	3
*Klebsiella pneumoniae*	15	21
*Proteus mirabilis*	12	17
*Providencia stuartii*	2	3
*Pseudomonas aeruginosa*	9	13
*Serratia macalane*	2	3
*Serratia marcescens*	5	7
*Serratia plymuthica*	2	3
**Total**	**70**	**100**

**Table 6:** Antibiogram pattern of *Klebsiella pneumoniae*, the predominant isolate recovered from CAUTI patients.

Antibiotics	Sensitive *n* = 15	Sensitivity %	Resistant *n* = 15	Resistance %
Amikacin (30 µg)	15	100	-	-
Cefepime (30 µg)	10	66.7	5	33.4
Colistin (10 µg)	13	86.7	2	13.4
Gentamicin (10 µg)	13	86.7	2	13.4
Ciprofloxacin (5 µg)	8	53.4	7	46.7
Levofloxacin (5 µg)	10	66.7	5	33.4
Meropenem (10 µg)	10	66.7	5	33.4
Ceftazidime (30 µg)	8	53.4	7	46.7
Ceftriaxone (30 µg)	3	20	12	80
Imipenem (10 µg)	13	86.7	2	13.4
Piperacillin/tazobactam (100/10 µg)	10	66.7	5	33.4
Teicoplanin (30 µg)	8	53.4	7	46.7
Cefoxitin (30 µg)	13	86.7	2	13.4
Nitrofurantoin (300 µg)	5	33.4	10	66.7
Ertapenem (10 µg)	13	86.7	2	13.4
Aztreonam (30 µg)	5	33.4	10	66.7
Amoxicillin/clavulanic acid (20/10 µg)	8	53.4	7	46.7
Sulfamethoxazole/trimethoprim (1.25/23.75 µg)	10	66.7	5	33.4
Mupirocin (200 µg)	3	20	12	80
Cefuroxime (30 µg)	5	33.4	10	66.7
Cephalothin (30 µg)	5	33.4	10	66.7
Tigecycline (15 µg)	3	20	12	80

**Table 7:** Antibiogram pattern of remaining bacterial isolates from CAUTI patients.

**Antibiotics**	***S. marcescens*** **(*n* = 5)**	***E. coli*** **(*n* = 7)**	***E. faecalis*** **(*n* = 9)**	***P. mirabilis*** **(*n* = 12)**	***P. aeruginosa*** **(*n* = 9)**
Amikacin (30 µg)	5	7	-	10	9
Cefepime (30 µg)	5	5	-	2	9
Colistin (10 µg)	3	7	-	2	9
Gentamycin (10 µg)	5	7	-	-	7
Ciprofloxacin (5 µg)	3	5	5	-	9
Levofloxacin (5 µg)	5	5	-	2	5
Meropenem (10 µg)	5	7	-	6	5
Ceftazidime (30 µg)	5	5	2	-	9
Ceftriaxone (30 µg)	3	-	-	-	3
Imipenem (10 µg)	3	7	-	-	7
Piperacillin/tazobactam (100 µg/10 µg)	5	-	-	10	2
Teicoplanin (30 µg)	-	5	2	-	2
Cefoxitin (30 µg)	2	7	-	12	2
Nitrofurantoin (300 µg)	-	2	6	12	-
Ertapenem (10 µg)	3	7	-	10	-
Aztreonam (30 µg)	5	2	-	-	2
Amoxicillin/clavulanic acid (20/10 µg)	-	2	6	7	-
Sulfamethox/trimethoprim (1.25/23.75 µg)	5	5	6	2	2
Daptomycin (30 µg)	-	-	9	2	-
Linezolid (30 µg)	-	-	6	2	-
Mupirocin (200 µg)	-	-	-	5	-
Tetracycline (30 µg)	-	-	5	2	-
Vancomycin (30 µg)	-	-	7	2	-
Erythromycin (15 µg)	-	-	-	2	-
Ampicillin (10 µg)	2	-	7	-	-
Moxifloxacin (5 µg)	-	-	7	-	-
Piperacillin/tazobactam (TZP) (36 µg)	-	-	-	2	2
Fusidic acid (10 µg)	-	-	2	-	-
Cefotaxime (30 µg)	3	-	-	-	-
**Antibiotics**	***P. stuartii*** ***(n =* 2)**	***A. baumannii*** ***(n* = 5)**	***K. oxytoca*** ***(n* = 2)**	***S. macalane*** ***(n* = 2)**	***S. plymathica*** ***(n* = 2)**
Amikacin (30 µg)	2	5	-	2	-
Cefepime (30 µg)	2	-	-	2	-
Colistin (10 µg)	-	3	2	2	-
Gentamicin (10 µg)	-	3	2	2	-
Ciprofloxacin (5 µg)	-	3	-	2	-
Levofloxacin (5 µg)	-	-	-	2	-
Meropenem (10 µg)	-	-	-	2	-
Ceftazidime (30 µg)	2	-	-	2	-
Ceftriaxone (30 µg)	-	-	-	-	-
Imipenem (10 µg)	-	-	-	-	2
Pipiracillin/tazobactum (100/10 µg)	-	-	-	2	2
Teicoplanin (30 µg)	-	-	-	-	-
Cefoxitin (30 µg)	2	-	-	-	2
Nitrofurantoin (300 µg)	-	-	-	-	-
Ertapenem (10 µg)	2	-	-	2	2
Aztreonam (30 µg)	2	-	-	-	-
Amoxicillin/clavulanic acid (20/10 µg)	-	-	-	2	-
Sulfamethoxazole/trimethoprim (1.25/23.75 µg)	2	-	-	-	2
Cefuroxime (30 µg)	2	-	-	-	-

## Data Availability

The original and raw data used and reported in this study are available from the first author and corresponding author.
